# Contributions to Power Grid System Analysis Based on Clustering Techniques

**DOI:** 10.3390/s23041895

**Published:** 2023-02-08

**Authors:** Gheorghe Grigoraș, Maria Simona Raboaca, Catalin Dumitrescu, Daniela Lucia Manea, Traian Candin Mihaltan, Violeta-Carolina Niculescu, Bogdan Constantin Neagu

**Affiliations:** 1 Department of Power Engineering, “Gheorghe Asachi” Technical University of Iasi, 700050 Iasi, Romania; 2 National Research and Development Institute for Cryogenic and Isotopic Technologies—ICSI Rm. Vâlcea, Uzinei Street, No. 4, P.O. Box 7 Râureni, 240050 Ramnicu Valcea, Romania; 3 Faculty of Electrical Engineering and Computer Science, Stefan cel Mare University of Suceava, 720229 Suceava, Romania; 4 Doctoral School Polytechnic, University of Bucharest, 060042 Bucharest, Romania; 5 Department Telematics and Electronics for Transports, University “Politehnica” of Bucharest, 060042 Bucharest, Romania; 6 Faculty of Civil Engineering, Technical University of Cluj-Napoca, Constantin Daicoviciu Street, No. 15, 400020 Cluj-Napoca, Romania; 7 Faculty of Building Services Engineering, Technical University of Cluj—Napoca, Bd. 21 Decembrie 1989, No. 128-130, 400604 Cluj-Napoca, Romania

**Keywords:** smart grid, clustering techniques, pattern clustering, power distribution planning, regression algorithms

## Abstract

The topic addressed in this article is part of the current concerns of modernizing power systems by promoting and implementing the concept of smart grid(s). The concepts of smart metering, a smart home, and an electric car are developing simultaneously with the idea of a smart city by developing high-performance electrical equipment and systems, telecommunications technologies, and computing and infrastructure based on artificial intelligence algorithms. The article presents contributions regarding the modeling of consumer classification and load profiling in electrical power networks and the efficiency of clustering techniques in their profiling as well as the simulation of the load of medium-voltage/low-voltage network distribution transformers to electricity meters.

## 1. Introduction

A few of the changes and problems the world’s population is currently facing are related to the climate, electricity, food, water, transport, utilities, health, education, administration, and industry. Cities use 75% of the energy produced and are answerable for 80% of all dioxide emissions, although they only cover 2% of the planet’s surface. Future cities will need to adapt in order to counteract the effects of factors such as environmental change, population expansion, and social mobility, together with migration, human conflicts and unfairness, economic globalization, technological advancements, food, water, and energy vulnerability, geostrategic shifts, etc. Future cities must manage infrastructure and resources more intelligently to meet people’s needs both now and, in the future, as the world becomes increasingly “urban” [1,2]. 

Mobility is at the core of modern society when the aforementioned factors are considered. Over the next 20 years, there will be a lot of changes in this area as global auto markets and the transportation industry are reshaped by electrification, shared mobility, vehicle networking, and autonomous vehicles. This transformation is supported by technological advancement and other crucial variables, such as legislative directives pointing these two sectors in the direction of low-carbon solutions and increased fuel efficiency [3,4,5,6]. 

In the medium and long term, automakers and major EV operators are ensuring that the decarbonization goals are treated to a higher standard. Around the world, there are currently more than 7 million electric vehicles (EVs) in use, and other aspects of road transportation, such as the freight industry, are being electrified.

The electric vehicle is not a recent invention; it first emerged alongside internal combustion engine vehicles. Electric cars outperformed all other car types between 1890 and 1900. Because they were less noisy and polluted than cars with internal combustion engines, they significantly increased in popularity at the beginning of the 20th century. However, the short battery life of electric automobiles meant that their owners could travel great distances.

In recent years, technological advancements and concerns related to climate change have progressively sparked the resurgence of electric automobiles. The transformation from internal combustion to electric engines, which is now occurring in the automotive area, represents the most significant change. To adjust to changing market conditions, automakers have undertaken massive financial investments and unforeseen alliances [7,8,9].

EVs have been more and more popular in recent years due to their capacity to provide a variety of benefits, including [9]:

- Energy efficiency: Electric vehicles use less energy than vehicles powered by traditional internal combustion engines (ICEs).
- Electric mobility improves energy security because the road transportation industry is so reliant on petroleum-based fuels. Additionally, electricity may be created using a range of materials and fuels and is frequently produced locally.
- Air pollution: Since electric vehicles produce no emissions, they are an excellent solution to the issue of air pollution, particularly in densely populated regions and those nearby, where many citizens can be exposed to dangerous toxins from transit vehicles.
- Greenhouse gas (GHG) emissions: Combined with a progressive increase in the production of low-carbon energy, increasing electric mobility can result in considerable reductions in GHG emissions from the transportation infrastructure when compared to other traditional vehicles. Additionally, electric vehicles can behave according to the integration of renewable energy, which is often unpredictable for generating electricity, and offer flexible services for power systems.
- Noise reduction: Electric vehicles, especially those with two or three wheels, are quieter than ICE vehicles.

Industrial advancement: Given the relevance of energy storage for the switch to “clean” electricity, electric vehicles also have the capability of storing generated energy. In essence, battery technology, one of the fundamental factors in industrial competitiveness, is a possible facilitator for the significant reduction in cost in the electric car industry.

In many cities across the world today, one can find personal automobiles, common transport, car sharing, taxis, municipal parking lots, two- and three-wheeled machines (mainly electric scooters), as well as an expanding number of commercial and freight vehicle sectors. The proposed study topic fits within the background and is related to actual policies in the decarbonization of transportation areas, minimization of urban pollution, and the integration of EVs into electrical systems [10,11,12]. 

This study’s objectives were to analyze the impact and potential effects of integrating many EVs into the power network, as the efficiency could be influenced in both positive and negative ways by the need to charge all these EVs, as well as suggest several strategies and measures to integrate them. The article discusses the effects of integrating electric vehicles into the electric distribution networks and analyses those effects from a technical standpoint in terms of how they affect urban electric distribution networks and how that affects changes in electricity usage. 

One challenge is connected to energy management, the impact of which may be reduced if the charging of EVs is achieved outside the peak period, which is likely to occur when big fleets of electric vehicles are incorporated into power networks. Electric vehicles (EVs) can be viewed from the perspective of electrical networks as either basic tasks (with continuous consumption) or using the Vehicle-to-Grid (V2G) idea, where storage devices plan recharging intervals or they can inject grid energy using their energy storage devices [9,10]. In the case of a significant uptake of EVs, it will be important to coordinate their functioning both as a potential source of revenue and as a new aggregator that affects the electrical network by managing demand.

If EVs are gathered or aggregated, they can contribute to the balancing process by lowering grid usage or injecting electricity into the grid as needed by helping to coordinate the load.

The article is organized in the following way: a presentation of the clustering methods used in this study is first given; electrical load profiling, load-type profiles correlated with low voltage (LV) consumers, and a distribution network loading simulation are discussed along with the results obtained, and then the conclusions are presented.

In recent years, technological developments in the energy domain, including the introduction of smart meters and the transition to the concept of “Smart Grids”, have provided transmission and distribution operators with opportunities to forecast the load required by the system, modeling consumers to take into account their behavior, for the prevention of unplanned outages, the optimal load planning of generating units, etc. [13]. In these cases, operators must manage a lot of data and perform complex analyses in order to make the best decisions regarding the optimal planning and operation of electricity networks. For the efficient management of large databases, there are two aspects that need to be considered: data extraction (data analysis to obtain specific knowledge, patterns, or models) and database management (data storage, processing, and querying). Both related concepts are crucial in the energy networks’ decision-making process [14]. The choice of technology and the organization of installation, processing, and maintenance operations are the first steps in the adoption of smart metering systems. These technologies mark a significant development in the interaction between users and network operators. If consumer-mounted smart meters were totally integrated into a modern metering infrastructure and data were adapted, then distribution operators (ODs) could have access to full monitoring, which would make it easier to estimate the state of distribution networks [15,16,17].

The transition to the “Smart Grids” concept can lead to the implementation of smart monitoring and remote communications equipment necessary for optimal power systems operation and planning that will lead to maximizing economic benefits and minimizing the environmental impact. The phrase “Smart Grids” represents a hyperbole that involves the management of the EEA without the intervention of the human factor. The key components of this idea include two-way communication with consumers and all other market participants, as well as digital control of the energy transmission and distribution network. This smart infrastructure will enable various energy services, markets, integrated distributed energy sources, and control systems. The world economy in the future will be supported by the smart grid. It suggests that, in many respects, electricity generation, transmission and distribution companies, regulators, and institutions, indeed, all levels of government, face a real challenge in terms of the energy sector which is the driving force behind the world economy [18,19,20,21,22].

Starting from the aspects highlighted above, the efficient solution to many problems related to the management of the electric power system goes through the elaboration of solutions based on one form or another of artificial intelligence. Artificial Intelligence (AI) techniques aim to create intelligent computing systems, systems based on the characteristics of human intelligence: reasoning, ability to learn, solve and communicate, systems for problems for which there is no classical computational algorithm. Over time, with the development of AI techniques, hybrid algorithms have been developed and perfected, such as fuzzy logic-controlled neural networks, fuzzy genetic algorithms or expert systems, and artificial neural networks generated by genetic algorithms or neuro systems, having already proven their effectiveness [23,24,25].

Because an AI system has more complex tasks to solve, the knowledge that needs to be represented in it increases (facts, rules and heuristics of the field, general concepts, and theories). In general, a system may work well, in line with the goal set by the knowledge provided, but any move outside its competence causes its performance to decline rapidly. This phenomenon is also called the fragility of knowledge [26].

Several machine learning techniques are presented in the studied literature, including supervised and unsupervised approaches that have been utilized for energy consumption level predictions. Among the unsupervised learning techniques, clustering is considered one of the most frequently applied techniques in data mining and machine learning. Clustering involves partitioning objects with similar patterns under observation into different groups. A vast number of works on clustering electricity usage patterns have been presented by researchers. The article [27] generated a typical load profile from data measured with automatic meter reading systems, then performed cluster analysis using three clustering algorithms, specifically, the hierarchical, k-means, and fuzzy c-means algorithms. In [28], classified daily load curves in industrial parks, which can be regarded as microgrids from the energy network perspective using SOM, then exploited k-means to obtain a number of clusters, and [29] demonstrated the possibility of applying disaggregation techniques on smart meter data via fuzzy c-means clustering. Similar work is in [30], where they utilized the k-means algorithm to group residential houses with similar hourly electricity use profiles, and in [31] where they proposed a method to characterize medium voltage electricity consumers by using several clustering algorithms. In order to choose the best one among the typical load profiles, they measured the performance of the clustering algorithms in terms of eight clustering validity indices. To deal with the scalability and computational complexity of the power consumption profiling process, the authors of [32] proposed a multi-layered clustering method for power consumption profiling. First, they acquired local power consumption profiles using k-means, considering clusters with a low number of patterns as abnormal power consumption behavior. In the second stage, a global power consumption profile was derived from the local ones. Furthermore, Refs. [33,34] applied an improved k-means algorithm with particle swarm optimization (PSO) to open residential buildings datasets to divide their electricity consumption in an entire region into different levels. The authors of [35] developed a methodology in which one-dimensional time series smart meter data were reshaped to two-dimensional arrays called load profile images. After performing image processing techniques on those images, they derived the class load image profiles via clustering algorithms. In addition, [36] partitioned customers into electricity user groups based on similar electricity usage behavior with the SOM, k-means, and hierarchical clustering algorithms. Similar to group electricity consumption profiles, the authors of [37] investigated a shape-based clustering method.

The discussed literature reveals several limitations of the employed techniques from various perspectives of energy consumption prediction. The literature lacks focus on capturing the recognizable patterns in building smart sensing data, which has a limited number of features. These features can be represented in low-dimensional feature space and may affect the overall performance of data analytic tasks. Many of the existing techniques enquire about the number of clusters to differentiate among distinct categories of data. In addition, the presentation of energy consumption for data analysts and common individuals is a common problem that has not been tackled effectively in the existing literature.

Therefore, based on the above-mentioned problems in household energy predictions, this paper presents a new framework with the following main contributions:

- There is not a lot of energy consumption data obtained from smart sensors for residential buildings, and the presence of missing data is a difficult problem in statistical analysis. Less than 1% of missing data can be considered a common problem, and between 1 and 5% can be considered a solvable problem. If the percentage is greater than 5%, difficulties may arise in solving the respective problems. Thus, if the values are between 5 and 15%, the problem requires the application of sophisticated solution methods, and if the value exceeds 15%, the problem may have difficulties in interpretation. Finding recognizable patterns in such data is very difficult, which affects the performance of electricity consumption analysis. To solve this problem, five hierarchical clustering methods were used in the clustering process: average distance (Average Method), the center of gravity (Centroid Method), minimum distance (Single Linkage Method), maximum distance (Complete Linkage Method), and Ward, which were the basis for obtaining the load-type profiles presented in the article. Based on the clustering methods used, we propose a method based on load-type profiles which is more robust, reduces calculation errors for off-peak time/peak time, requires less computation volume, and converts small model representations from data with reduced dimensions in high-level representations, comparable to using large databases.
- Clustering algorithms require an input parameter to divide data into multiple clusters. This article, by using the method of simultaneous layers in the hierarchical clustering process, average distance (Average Method), the center of gravity (Centroid Method), minimum distance (Single Linkage Method), maximum distance (Complete Linkage Method), and Ward, obtained an adaptive grouping to organize large data.
- After dividing the data into several clusters, the regression model based on first- and second-degree polynomials for every consumption class was applied, performing a predictive statistical analysis on the data to determine which buildings had a high, medium, and low level of energy consumption.

## 2. Materials and Methods

### 2.1. Clustering Techniques

**Figure 1:**
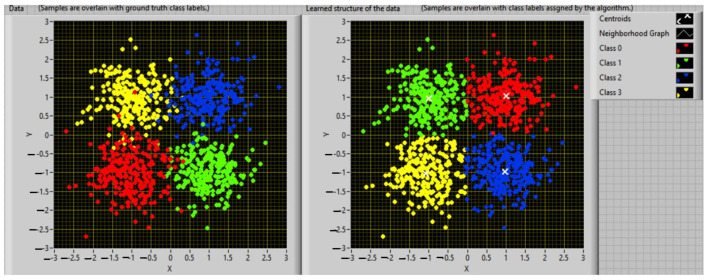
Example of clustering processing.

The use of clustering algorithms allows for the spatial distribution of characteristic vectors to be used as a basis for grouping input data. Each element connected with a set of data will be characterized by a vector whose components are represented by the representative qualities or attributes of the vector in order to examine the similarity or differences between them and to categorize them. The determination of the characteristic/attribute number and their definition requires a deeper analysis of the database designed on the available information and considering the expertise of specialists [38,39]. The vectors connected to the input data are grouped throughout the clustering phase based on the estimated distance between each of them. Depending on the analyzed topic, the clustering process will result in one or more clusters (groups, patterns, models, or classes), which describe the spatial position of the qualities taken into account for the process’ elements. Within each cluster, the elements are closer to a common center when compared to other centers belonging to other groups. This aspect is exemplified in Figure 1.

In Figure 1, the elements, represented by vectors with two characteristics $\left(x_{i},y_{i}\right)$, *i* = 1,…, *N* (where *N* is the maximum number of elements of the database subjected to the clustering procedure) were assembled using a similarity principle defined by the distance calculated between vectors. In this mode, two or more components can be associated with the same pattern if the distance between them is smaller in relation to the distance from the elements of another cluster. Finally, each cluster will be characterized by a representative element, determined by mediating the characteristics of the elements that make up the cluster [40].

### 2.2. Stages of the Clustering Procedure

Clustering processes can be applied in various domains in order to group unlabeled components. These domains already involve various assumptions, terms, or techniques, related to clustering procedure phases, as a function of the addressed problems.

**Figure 2:**
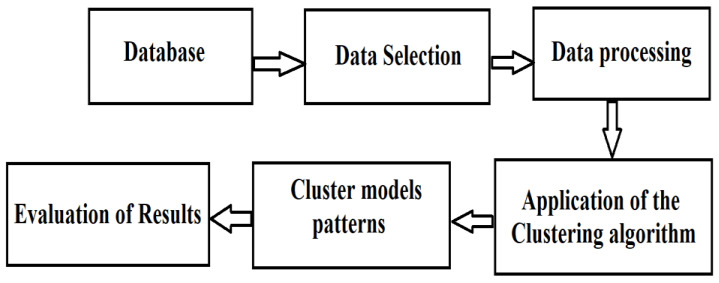
Clustering process phases.

The steps that must be covered are described below and represented in Figure 2 [40,41].

Step 1. The components subjected to the clustering procedure must be established. In this phase, it is necessary to consider the option from the database that is best suited to the aim of the problem. The type and size of the attributes available for the clustering procedure can be chosen.

Step 2. The attributes/characteristics of the components subjected to the process must be extracted. The identification of the most useful and important characteristics must be achieved. During this process, one or more component transformations can be accomplished to obtain new dominant attributes.

Step 3. A similarity measure must be defined. Usually, the similarity can be determined by measuring the distance connecting adjacent items. Once a vector has been attributed to each component, this length may indicate how similar two elements are. The literature has defined several different distance measurement techniques, with Euclidean distance being the most used.

Step 4. This phase represents the actual clustering procedure. It can be achieved in various modes depending upon the techniques chosen by the decisional factor. All clustering techniques should conduct several clusters for any input data set. If no clusters resulted from the process, other techniques can be applied in order to obtain the desired results. The obtained results can be “clear”, meaning that the separation of the components is achieved in well-defined clusters, or they can be “fuzzy”, meaning that each component has a degree of dependence on each cluster.

Step 5. The results must be extracted. For this, an accurate interpretation of the results is necessary so that their rendering can be achieved in a simple way that is easy to interpret by the decisional factor. In this scenario, either from the point of view of automatic analysis (where a computational system can perform further data processing effectively) or from the perspective of a human, simplicity is required (the representation used for results is easier to understand by decision makers). The extraction of the results from the clustering method is a brief illustration of each cluster using representative components.

Step 6. Evaluation of results. An assessment of the clustering procedure is taken into account when analyzing the validity of the outcomes (represented by clusters), and this evaluation often employs an optimization criterion. This impartial analysis examines whether the outcomes are accurate. If a cluster does not occur unintentionally or for other reasons, it is validated.

### 2.3. Clustering Methods

Next, certain terms will be defined and the notions that will be used will be briefly described in the next section.

The element $x^{j}$, *j* = 1,…, *N* (where *N* is the total number of elements), is a unique object used in the clustering process. It is usually represented by an n-dimensional vector $x^{j}=\left[x_{1}^{j}\;x_{2}^{j}\;\ldots \ldots x_{n}^{j}\right]$.

Scalar components $x_{i}^{j}$, *i*, *i* = 1,..., *n*, are called characteristics/attributes of the vector $x^{j}$ and are established by the decision maker.

The distance between vectors is a metric in the space of the attributes $x_{i}^{j}$, *i* = 1, .., *n*, corresponding to input vectors $x^{j}$, *j* = 1,…, *N*, and used to determine the similarity between elements. The most used is the Euclidean distance.

For a database X, consisting of the vectors $x^{j}$, *j* = 1,..., *N*, with n characteristics, $x^{j}=\left[x_{1}^{j}\;x_{2}^{j}\;\ldots \ldots x_{n}^{j}\right]$, different distances between vectors can be defined. Thus, if two vectors $x^{r}$ and $x^{s}$ are taken into account, the distance can be calculated with the relation:

(1) $$d\left(x^{r},x^{s}\right)=\sqrt{\left(x^{r}-x^{s}\right){\left(x^{r}-x^{s}\right)}^{t}}$$

There are several ways to classify clustering methods in the literature. The most used classifications are given in [40,42]: hierarchical methods and the K-means method.

Hierarchical clustering methods can be subdivided, according to their meaning, into methods of agglomeration and division. In the case of agglomeration methods, for example, we start from the k clusters, each containing a single element $x^{j}$, *j* = 1,..., *N*, and by successive mergers, form a single cluster, containing all N elements. In the case of division methods, the direction of deployment is inverse, i.e., starting from a single cluster containing all the $x^{j}$, *j* = 1,..., *N* elements, we reach *k* clusters, each containing a single element $x^{j}$. Agglomeration techniques are usually used more frequently. As shown above, in the hierarchical spatial grouping, an agglomeration process goes through a series of mergers/couplings of groups/classes, $P_{n},\;P_{n-1},\;\ldots .,\;P_{1}$. The first, $P_{n}$, consists of *n* “groups” with a single element/object, and the last $P_{1}$ includes a single group having all n elements/objects. At each stage, the method couples two close groups (at the first level, of course, this means the coupling of two elements/objects that are close to each other (in distance), since at the initial stage, each group has an element) [40,43].

The clustering process can be illustrated as a two-dimensional diagram named a dendrogram. These methods are suitable for small tables, having a few hundred rows. The desired number of clusters can be chosen after the proper shaft is designed by imposing a threshold [30,40].

The difference among the agglomeration techniques is given by the method for defining the distance between the clusters.

Considering two clusters, $C_{r}$ and $C_{s}$, containing $n_{r}$ and $n_{s}$ elements, the average distance $d\left(C_{r},C_{s}\right)$ is calculated on an Euclidean distance base:

(2) $$d\left(x_{r}^{f},x_{s}^{h}\right)=\sqrt{\sum_{k=1}^{N}{\left(x_{rk}^{f},x_{sk}^{h}\right)}^{2}}$$

can be expressed through the relation:

(3) $$d\left(C_{r},C_{s}\right)=\frac{1}{n_{r}n_{s}}\sum_{f=1}^{n_{r}}\sum_{h=1}^{n_{s}}d\left(x_{r}^{f},x_{s}^{h}\right)$$

Starting from the above-mentioned, the most used hierarchical clustering methods are briefly presented below.

**Figure 3:**
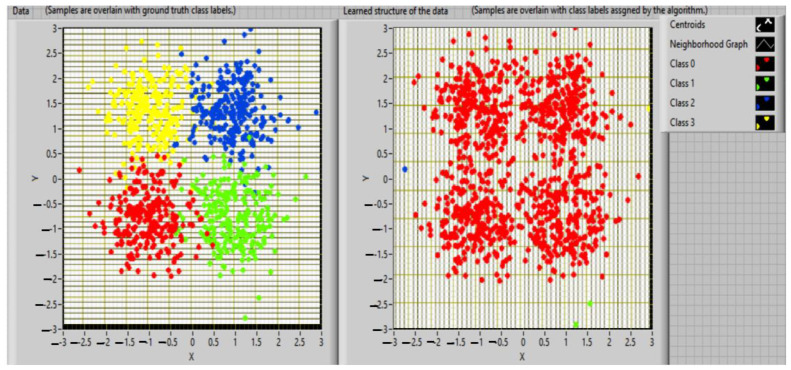
The representation of hierarchical clustering algorithms.

The following figure shows the result obtained by applying the hierarchical clustering algorithm (Figure 3).

a. Minimum distance method (minimum method)

**Figure 4:**
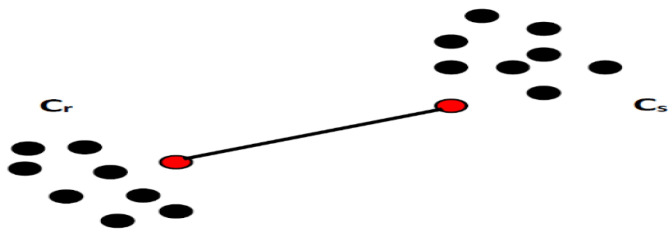
A representation of the minimum distance between two clusters.

This method is the simplest, being based on the minimum distance, also known as the method to the closest neighbor. In such cases, the distance between the clusters represents the distance between the closest items:

(4) $$d\left(C_{r},C_{s}\right)=min\left\{d\left(f,h\right)\right\}$$

where the *f* component is attributed to the $C_{r}$ cluster and the element *h* to cluster $C_{s}$. In this case, the distance is calculated between each possible pair of elements (*f*, *h*). The minimum value is the distance between the cluster’s $C_{r}$ and $C_{s}$. In other words, the distance between two clusters is given by the shortest link value. At every stage of the clustering process, the $C_{r}$ and $C_{s}$ clusters, for which $d\left(C_{r},C_{s}\right)$ is minimal, will be coupled. A graphical interpretation of the minimum distance between the clusters is shown in Figure 4.

b. Maximum distance method (maximum method)

**Figure 5:**
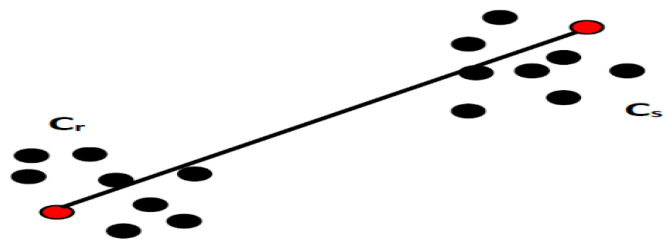
A representation of the maximum distance between two clusters.

This method, also known as the farthest neighbor method, is based on distance maximum, being the opposite of the minimum method. In this technique, the distance $d\left(C_{r},C_{s}\right)$ is calculated using the equation:

(5) $$d\left(C_{r},C_{s}\right)=max\left\{d\left(f,h\right)\right\}$$

where the *f* element is attributed to the $C_{r}$ cluster and the *h* element to the cluster $C_{s}$. In such a case, the distance between two clusters is given by the longest link value. At each phase of the spatial hierarchical grouping, the clusters $C_{r}$ and $C_{s}$, for which $d\left(C_{r},C_{s}\right)$ is maximum, will be coupled. Figure 5 presents a graphical interpretation of the distance between the clusters.

c. The average distance method

In this method, the distance between two clusters is defined as the average of the distances between all element pairs, where each pair contains one element from each cluster. The average distance $d\left(C_{r},C_{s}\right)$ is calculated with the relation:

(6) $$d\left(C_{r},C_{s}\right)=\frac{T_{r,s}}{\left(n_{r}\times n_{s}\right)}$$

where $T_{r,s}$ is the sum of all possible distances between the elements of $C_{r}$ cluster and the elements of $C_{s}\mathrm{cluster}$ and *n_r_* and *n_s_* represent the number of elements in the cluster $C_{r}$ and $C_{s}$, respectively.

**Figure 6:**
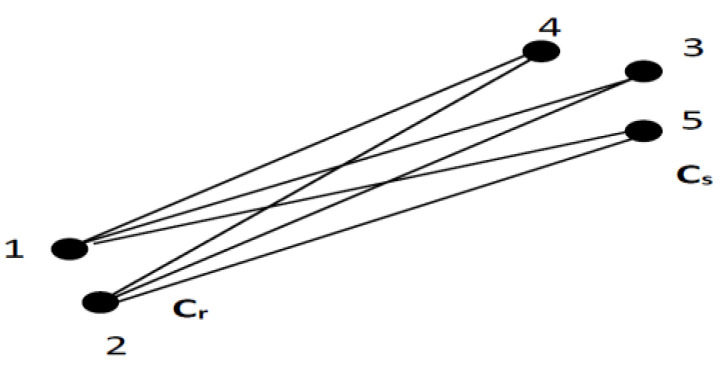
Representation of the average distance between two clusters.

At each stage of the clustering process, the $C_{r}$ and $C_{s}$ clusters for which the distance $d\left(C_{r},C_{s}\right)$ is minimum, are coupled. Figure 6 illustrates how the average distance is defined.

d. Center of weighted method

In this technique, the distance between two clusters is described as the square Euclidean distance between their centers of weight.

(7) $$d\left(C_{r},C_{s}\right)={\left\|\bar{x_{r}}-\bar{x_{s}}\right\|}^{2}$$

where $\bar{x_{r}}$ and $\bar{x_{s}}$ are the mean vectors for the $C_{r}$ and $C_{s}$ clusters.

This method is much more robust, deviating from the average more than other methods of hierarchical clustering, but in other situations may not give as good results as the Ward method or the average distance method.

e. Ward method

This method seeks to form $P_{n},\;P_{n-1},\;\ldots .,\;P_{1}$ partitions in a way that minimizes the information loss associated with each cluster and measures them in an easy-to-use interpreted form. At each step of the analysis, two clusters are combined, the fusion of which leads to results that minimize the increase in “lost information”. The lost information is defined by Ward in the conditions of the criterion of the square sum of the error.

(8) $$d\left(C_{r},C_{s}\right)=\frac{{\left\|\bar{x_{r}}-\bar{x_{s}}\right\|}^{2}}{\frac{1}{n_{r}}+\frac{1}{n_{s}}}$$

where $\bar{x_{r}}$ and $\bar{x_{s}}$ are the mean vectors for the $C_{r}$ and $C_{s}$ clusters and *n_r_* and $n_{s}$ represent the number of elements from the $C_{r}$ and $C_{s}$ clusters, respectively.

f. *K*-means method

This method involves a simple and easy mechanism to classify the input data set into several *K* clusters (*K* fixed a priori). The basic plan implies defining *K* centers of *weight*, one by one for each group. These centers of *weight* must be rationally fixed because different locations lead to different results. The best choice is to fix them, if possible, as far apart as possible from each other. The next step is to take each element of the input data set and link it with the closest center of *weight*. The first grouping stage ends when there are no more ungrouped items. At this point, it is mandatory to recalculate new *K* centers of the groups arising from the previous phase. The process continues until the positions of the new centers no longer change significantly.

The objective of the method is to minimize an objective function (quadratic error function) given by the expression:

(9) $$J=\sum_{k=1}^{K}\sum_{l=1}^{n_{k}}\|x_{l}^{k}-c_{k}{\|}^{2}$$

where $\|x_{l}^{k}-c_{k}{\|}^{2}$ is the distance measured between point $x_{l}^{k}$, *l* = 1,..., *nk*, where $n_{k}$ is the total number of components of the k cluster, and the center of the group $c_{k}$, *k* = 1,..., *K*.

### 2.4. Validation of Results

The evaluation of the results obtained from the clustering process is the main concern of cluster validation. At this stage, the density, size and shape, separation of clusters, and robustness of classification were examined. The literature mentions the following tests to validate the clustering process [39,40,44,45]:

- External tests—data not included in the basic ranking are compared with the categorization results of the input data.
- Internal tests—only input data are utilized to evaluate the classification’s quality; each cluster’s separate validation is carried out using this test.
- Relative tests—this approach takes into account several classifications of the database, the results being analyzed using the same clustering method, but with various input data.

Internal cluster validation tests are more common and effective in real-world settings. Testing based on the creation of a global silhouette index of clusters is one of them and is also one of the most popular. This test determines the average shape width for each cluster, the median shape width of each element, and the average shape width of the entire collection of input data. With this method, each cluster might have a “shape” that is based on comparing its separation and density. The clustering procedure is then validated using the shape’s average width, and the ideal cluster number will also be set using the same information.

(10) $$GSI=\frac{1}{N_{k}}\sum_{k=1}^{K}F_{k}$$

$F_{k}$, the local silhouette coefficient, is calculated using the relation:

(11) $$F_{k}=\frac{1}{r_{k}}\sum_{l=1}^{r_{k}}f_{l}$$

$f_{l}$, the silhouette width coefficient for element *l*, is calculated by:

(12) $$f_{l}=\frac{b_{l}-a_{l}}{max\left\{b_{l},a_{l}\right\}}$$

$\mathrm{where}\;a_{l}$ is the average distance from element *l* and the elements from cluster *k* and $b_{l}$ is the minimum median distance from component *l* and the components in the closest cluster *k*.

In Equation (12), if the element *l* is unique inside a cluster, then $f_{l}$ = 0. The literature proposes the following explanation of the GSI coefficient [38,40]:

- 0.71 to 1.00 (a strong structure was highlighted);
- 0.51 to 0.70 (a reasonable structure was obtained);
- 0.26 to 0.50 (the structure is weak and might be artificial);
- <0.25 (no substantial structure was noticed).

Verifying the clustering process quality is one of the main steps in analyzing the inherent database characteristics. Its purpose is to evaluate the results of the process clustering and select the schema that best fits the elements in the database.

### 2.5. Electric Load Profiling

The loads from the nodes within the electrical networks range in consumption time and place. Consequently, distribution operators (ODs) need details regarding the load of fed consumers so that they will be able to optimally plan and operate the network and ensure proper power supply and operation modes, load management, and proper billing [40,46,47,48]. The load demanded by consumers depends on various parameters such as:

- Consumer type: consumption type, with/without electric heating, or size of the building;
- Time factor: time of day, weekday, and month;
- Climatic factors: humidity, temperature, cloudiness, wind speed, etc.;
- Other electrical charges related to the analyzed load.

For a certain consumer, his behavior is determined by a load profile correlated with the electricity consumption for each interval. The accessibility of this data is dependent on the type of consumer. In general, small consumers (such as residential ones) have an uncertain behavior, because the implementation of smart metering on a large scale would lead to large investments whose recovery time from their energy consumption would be too long. For these consumers, there is only the consumption of electricity at certain periods of time each year. For large consumers (such as industrial consumers), the installation of smart meters is facilitated by advantages related to billing (done every month) and high electricity consumption (justifying the investment by the fact that the detailed recording of consumption allows for the application of differentiated tariffs varying with consumption period).

In the traditional strategy for a distribution system plan, load profiles are employed to evaluate the maximum necessary load, in correlation with the simultaneity coefficient of the consumers coupled to the network node. Despite the fact that this strategy is appropriate, some major disadvantages arise:

- There are inherent inaccuracies, due to the simultaneity coefficients, which must be highlighted;
- The energy consumption and losses calculation does not have an increased precision;
- The voltage within the network nodes from various hours is not known;
- The load profiles of nodes having arbitrary variations in power requirement cannot be accurately modeled or evaluated.

Utilizing modern techniques of load analysis, load forecasts and the calculation of power and energy losses (for any period) can be achieved. The use of load profiles has some advantages [40,49]:

- It is not necessary to estimate or calculate concurrency coefficients, as load profiles already include the information;
- The energy consumption and power/energy losses calculation can be correctly achieved at any point within the network;
- The main voltage and charging are known for any period;
- The optimal position of the transformer plot can be determined, both for the peak load period, as well as for other times of the day;
- The effect of overloading or increasing the load is modeled more accurately than in the traditional method.

Medium voltage (MV) and low voltage (LV) networks, mainly urban ones, have many nodes even if only the nodes where electric distribution substations (EDSs) are located are taken into account. Thus, monitoring the system consumption for every node can become overwhelming, sometimes virtually impossible. This is practically overcome if, in the organization studies corresponding to the networks, the load-type profiles are associated with the node groups.

### 2.6. Load-Type Profiles Correlated with Nodes in Electrical Distribution Networks

This paragraph presents an approach based on hierarchical clustering techniques (presented in Section 2) for calculating the load-type profiles (LTP) correlated with nodes’ high voltage (HV) and medium voltage (MV) electrical distribution networks. Through knowledge of the load profile of the nodes, the OD can clarify the procedure for estimating the requirement in a certain sector.

In this respect, it is mandatory to understand the daily loading profiles. The load diagram of the nodes must be reconstructed by applying the standard load profile and daily demand. This standard profile is characterized by 24 or 48 load values.

The type and season influence the shape of load profiles. Because many profile responsibilities are correlated with various nodes of the network and may complicate the problem, they must be grouped into clusters, taking into consideration some similarities among them. For every cluster, the typical load profile can be established.

In this sense, all the measurements performed must be processed, by arrangement and normalization, using a convenient normalization factor (average power, peak power, or more frequently, the energy consumption of the studied period):

(13) $$p_{i}^{h}=\frac{P_{i}^{h}}{\sum_{h=1}^{T}P_{i}^{h}},i=1,\ldots \ldots ,N$$

where $p_{i}^{h}$ is the normal value of the power in *i* node at *h* hour, $P_{i}^{h}$ is the real value of the power in *i* node at *h* hour, and $\overset{T}{\underset{h=1}{\sum }}P_{i}^{h}$ is the total energy consumption in the interval *T* (24 h).

It is worth mentioning that after the clustering procedure is applied, clusters can be acquired as coherent and representative so that the diagrams within the same cluster are similar [40,49,50]. In the end, every cluster will be related to a typical task profile, estimated using the graphs median.

(14) $$m_{C_{k}}^{h}=\frac{\sum_{i=1}^{N_{C_{k}}}p_{i}^{h}}{N_{C_{k}}};h=1,\ldots \ldots ,24;k=1,\ldots ,N_{K}$$

where $\;N_{K}$ is the cluster numbers derived from the node’s classification in concordance with the absorbed load (active power) and $\;N_{C_{k}}$ is the node number from each cluster $C_{k}$, $k=1,\ldots ,N_{K}$.

### 2.7. Load-Type Profiles Correlated to Low-Voltage Consumers

In recent years, distribution operators have increasingly used smart metering systems (Smart Metering System) to monitor the electricity consumption of consumers. The development of these systems begins with technology selection and planning for installation, operation, and maintenance. In general, the implementation of residential or non-residential consumer categories is quite discrete in the case of many countries in the European Union. Currently, there are two alternative solutions to solve the problem of metering electricity consumption for consumers.

The first solution envisages the installation of smart meters for all consumers, being expensive and uneconomical, but the most accurate. The second solution envisages the continued use of traditional meters and the attachment of load-type profiles to the monthly energy consumption, which is then distributed over days and hours.

For large consumers, in the category of industrial ones, the presence of smart meters is necessary for several reasons: invoicing is done every month, the consumption of electricity is high (which implies rapid amortization of the investment), and it provides a detailed recording of consumption necessary for the process of invoicing.

This section presents an algorithm for determining the type of load profiles associated with LV consumers according to the category of energy consumption in which they fall. Consumption categories are identified from historical information and can be updated following changes in consumer behavior.

The algorithm phases are [40]:

Phase 1. In this phase of load analysis and database formation, a representative specimen must be identified from the crowd of consumers who have installed smart meters and the sampling step for the purchase of load schedules must be described. Consequently, a database with the registered load program and the consumer category is created.

Phase 2. Technical problems in the pre-processing of the data may affect the quality of the database in real cases of monitoring consumer charges, requiring many meters spread over a large geographic region over an extended period. The most relevant and frequent problems are communication issues, interruptions, meter failure, and, occasionally, the irregular behavior of individual consumers. These issues will influence the records of the database, appearing as null values or exceeding a particular threshold set by the connection notice. Such records must be identified and working techniques must be applied to obtain the missing data, resulting in the substitution of missing or equal to zero data with some estimated values so the database can be made ready to obtain clusters.

Phase 3. The database with records of load schedules must be divided into clusters of consumption, defined by the consumer’s type: residential, commercial, or industrial.

Phase 4. This phase represents the clustering procedure. A hierarchization of clusters is achieved, taking into consideration the daily consumers’ energy consumption within every consumption macro-category. In this respect, the K-media clustering technique is applied. For every cluster, the representative load profile is determined by applying the average of the load graphs’ hourly values.

Phase 5. Task-type profiles must be assigned. For every customer’s class, a typical task profile is assigned as a function of the activity macro-category to which the consumption belongs.

The suggested algorithm was evaluated using a database with 296 load diagrams. After the macro-categories were divided, 147 consumers were distributed in the residential consumers class, 97 in the commercial consumers class, and 52 in the industrial consumers class.

The utilization of the k-means clustering technique within every activity macro-category resulted in five clusters within the residential consumers class and three clusters within the commercial and industrial consumers ones.

**Figure 7:**
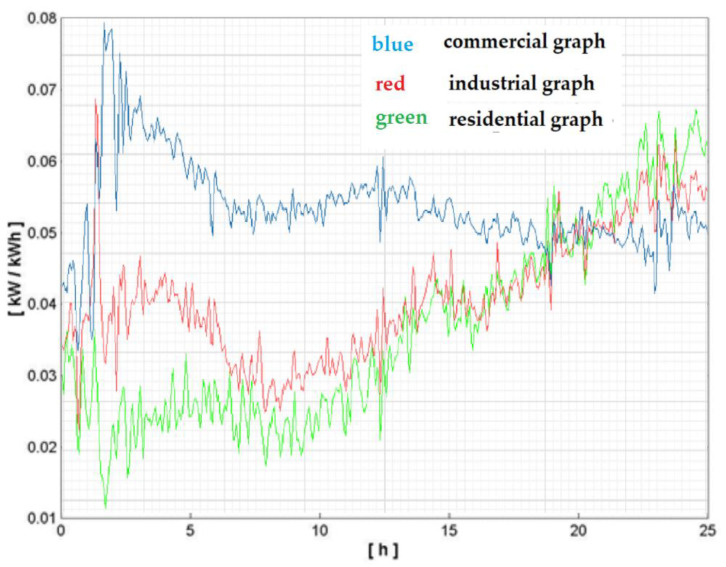
Load-type profiles of activity macro-categories.

Using these standard profiles, consumers can be better delineated in connection with load modification than the standard profiles correlated to the complete activity macro-category. This detail can be emphasized if a correlation is achieved between the load-type profiles of every activity macro-category and the load-type profiles linked to these macro-categories (Figure 7).

The change in consumers’ energy consumption determines important problems in the planning of activities associated with the technological procedure, in terms of adopting the optimal power supply and operation solutions. Solving these issues can be efficiently achieved by utilizing consumption profiles correlated to energy carriers.

This section proposes a perspective based on clustering methods for the determination of load-type profiles for electric vehicle charging networks. The standard profile’s forms, which take into account both the type of electrical equipment or installations supplied and the time of year the survey is conducted, represent the specifics of how consumers use electricity.

**Figure 8:**
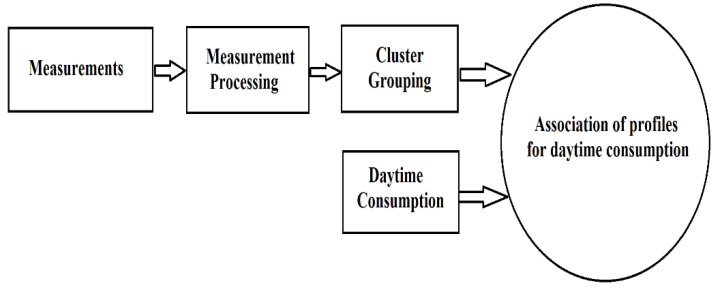
Scheme for the determination of load-type profiles in electric vehicles considered as industrial consumers.

The methodology proposed for the load profiling process for electrical vehicles as (industrial) consumers is presented in Figure 8 [40].

The importance of every phase from Figure 7 is the same as for all profiling procedures that are operated with clustering methods from the previous paragraphs.

### 2.8. Distribution Network Loading Simulation

In the distribution network, the basic component represents the loading simulation of MV/LV transformers. Because tens of thousands of transformers are positioned in the distribution matrix, their hourly load is hard to determine, due to the many distribution networks, including current and voltage sensors mounted in devices, transformers, and MV connections unequipped with recording meters with transmission capacity, remote, real-time recording, and load level. Consequently, it is hard to identify those transformers operating at overload or to estimate the loads of MV connections intended for transfer between distributors without a simulation.

The most effective route to evaluate a load of transformers, without performing real measurements, is represented by the utilization of simulation programs, with some of the following elements being taken into consideration [40]:

1. The number of consumers coupled to every transformer;
2. The consumer type;
3. The annual energy consumption for every consumer;
4. Task type diagrams correlated with every consumer class;
5. Software able to calculate the load of transformers.

It must be emphasized that, when a load of transformers is estimated, the maximum and hourly active powers (within low voltage/medium voltage side) are computed on peak days and varying typical intervals (winter, summer, average working days of the week, etc.).

### 2.9. MV/LV Distribution Transformers Load Simulation by Clustering Procedure

The database structure that is necessary to simulate the load of the distribution transformers in the MV/LV substations includes:

- The LV database having a “consumer link-substation (CS)” (number and class of consumers from every transformer in CS);
- Basic profiles containing task-type profiles for all consumers classes;
- A consumption database containing data on the annual energy consumption and consumer class.

**Figure 9:**
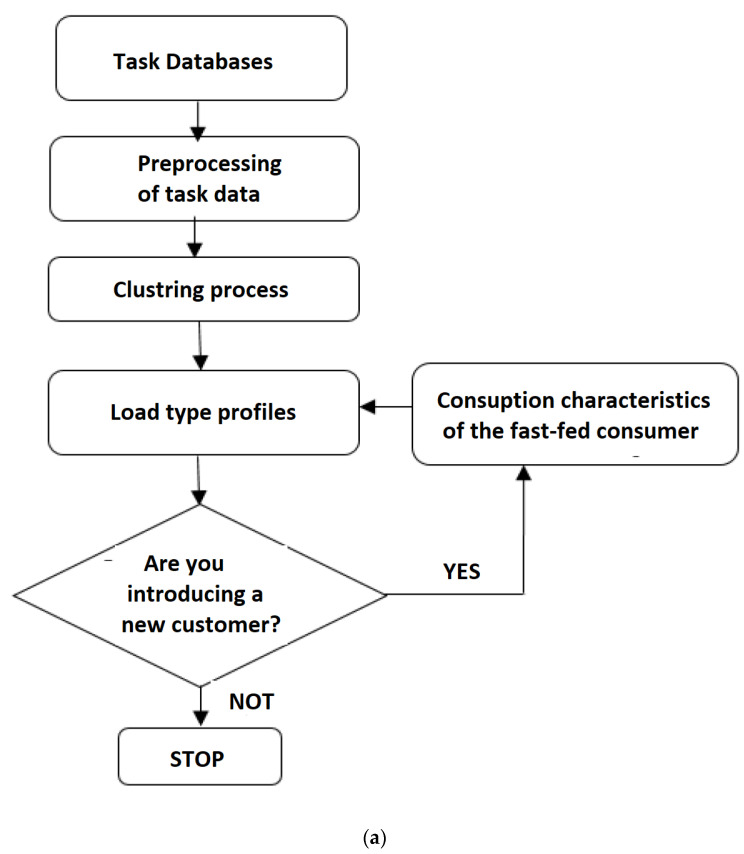

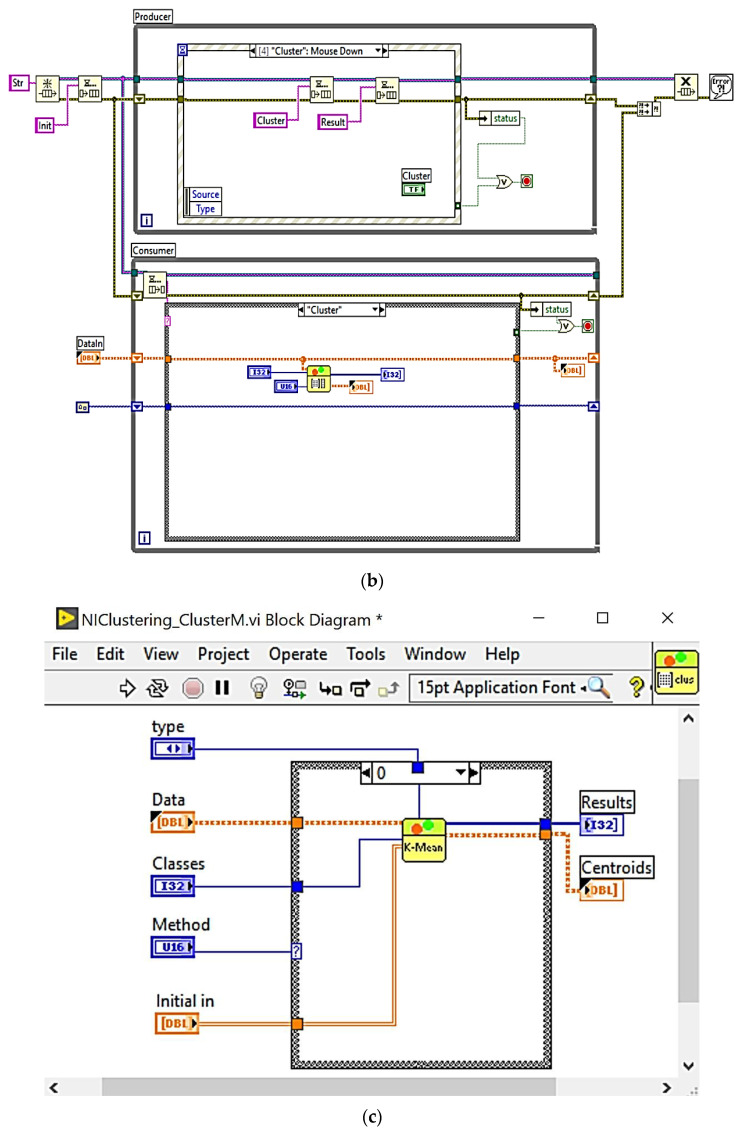

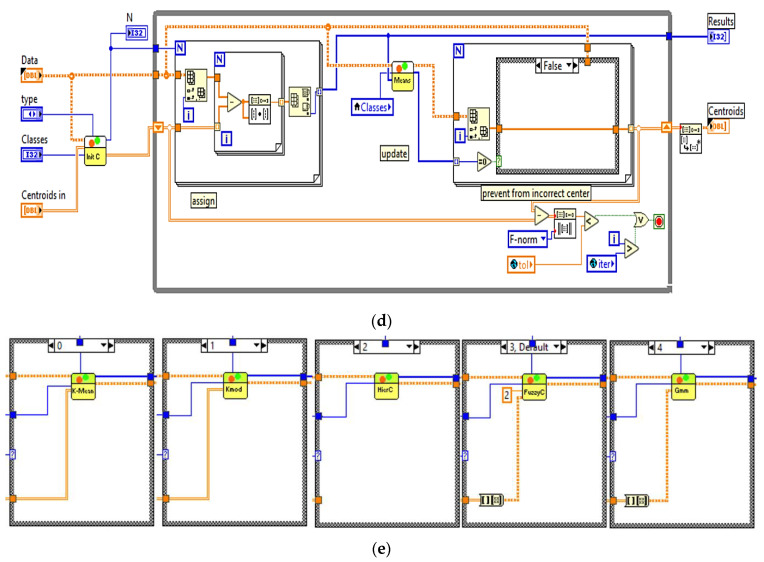
Implementation diagram for determining the load-type profiles for residential and non-residential LV (**a**) consumers and the software pseudocode: (**b**) is master code, (**c**) implementation code for k-means, (**d**) implementation code for hierarchical cluster and (**e**) represent software modules for all clustering methods.

In order to establish the typical load profiles correlated with consumers in LV networks, a database is required to include as many registered load diagrams as possible, representing all consumption categories. The decisional procedure of associating a typical task profile with a certain consumer constitutes a complicated problem. Consequently, a load profiling algorithm using clustering methods has been suggested for residential and non-residential consumers. Figure 9 shows the implementation diagram for determining load-type profiles for residential and non-residential LV consumers [40] and the software pseudocode. In the following figures, the software implementation for the proposed algorithm is represented. The software development was carried out in the LabVIEW programming language developed by National Instruments.

## 3. Results

An important issue in the optimal operation and planning of electric distribution networks by electric companies is the estimation of the maximum load of consumers. This problem occurs especially with home consumers. The decision to determine the optimal number of consumption categories within the same class of consumers and the estimation of the maximum load is a complex problem. Therefore, an improved regression method with clustering techniques is presented. Within the proposed method, to define the consumption categories of consumers, a classification can be made considering the monthly energy consumption and the maximum load (data obtained from the recorded load graphs). After identifying the consumption category, the maximum load of each consumer is estimated using a regression model corresponding to each consumption class. The major advantage of the proposed method is the exploration of the data using clustering techniques in order to obtain models/patterns/categories of consumption.

The contributions to modeling loads in electrical networks, the effectiveness of clustering techniques in their profiling, and the load simulation for MV/LV distribution transformers using clustering procedures are highlighted in the study.

### 3.1. Estimating the Maximum Load of LV Users (Consumers) Using Clustering Techniques

The estimation of the maximum load of users is a key parameter in the effective operation and planning of electricity distribution networks by energy providers. This problem occurs especially in household consumers, but also in the integration of electric vehicle charging. From analyzing the built databases and using data exploration techniques, load patterns/models can be identified that can be extrapolated to other potential consumers such as electric vehicle charging networks.

The decision to estimate the appropriate number of consumption categories within the same consumer class and the maximum burden is a complex issue. Thus, a customized regression method using clustering techniques is presented.

The monthly energy consumption, as well as the maximum load, can be classified using the suggested approach to determine the consumption groups of users (data obtained from the recorded load graphs). After determining the consumption category, a regression pattern matching every consumption class is used to estimate the maximum load of each consumer. The main benefit of the suggested approach is the data exploration utilizing clustering techniques to produce models, trends, and consumption categories that would aid in consumer modeling.

Its stages are presented below [40]:

Stage 1. Database. For consumers in the selected pilot area where smart meters have been installed, the load graphs for the analyzed time period are recorded. For each consumer, the variables that characterize the consumption category (maximum load, monthly and annual energy) are extracted.

Stage 2. Data pre-processing. All records from the database are analyzed, and those that contain missing values, equal to zero or atypical, are subjected to the process of treatment with missing data techniques. After all processing, the data are used to classify consumption categories.

Stage 3. Data exploration process. The first step in this stage is the use of clustering techniques in order to obtain consumption categories according to the maximum load and monthly energy consumption. The K-media technique is utilized for the clustering process. Then, a regression model is built for each consumption category to estimate the maximum load related to consumers.

Step 4. Estimation of peak load. The regression models obtained in Stage 3 are used to estimate the maximum load absorbed by the monitored consumers by means of classical meters.

The structure of the database includes information on the type of consumers (residential, industrial, commercial, and public) and their daily/monthly/annual consumption respective to the maximum load absorbed. This information can be obtained using smart meters. The information acquired is represented by the load curves that describe the consumer’s behavior during the day. The processing of the load curves allows us to determine the data on energy consumption respective to the maximum load absorbed by each consumer. For the method implementation, the database was split in two, a working base and a testing base. The application of the working base will lead to mathematical regression models able to conduct the estimation of the maximum load for each type of consumer within the same category of energy consumption. Energy consumption categories are then acquired using the K-average clustering procedure for each consumer type (residential, industrial, commercial, and public). The outcomes of the clustering technique are confirmed using a group quality assessment, based on the shape coefficient. The method is then tested to estimate the maximum load absorbed by consumers belonging to the testing base.

The method was applied using an initial database containing records of load schedules for 1160 household consumers located in a countryside region over a one-month period. For each consumer, the characteristic variables related to the peak load (P_max_) and monthly energy consumption (W_luna_) were extracted.

In Stage 2, the analysis of load graphs and characteristic variables led to the elimination of 15 consumers due to zero energy consumption. Thus, in the next phase, the database consisted only of the associated information for 1145 consumers. This base was split in two, a working and a testing base. The size of the working and testing bases varies in the literature and primarily depends on the total amount of records in the original database. The split between the two bases ranges from 90/10 to 60/40. In this instance, the working database contained 814 consumers (representing 66 percent of the database), leaving the testing database with the remaining customers (331 consumers, 33 percent of the database).

In Stage 3, the data exploration process was initialized through the K-media clustering method to obtain the consumption categories in which the consumers in the work base will be integrated.

The first phase of the K-average method consisted of the determination of the maximum cluster number (consumption categories) with the relation:

(15) $$C_{k\;max}=\sqrt{N}=\sqrt{841}=29$$

**Figure 10:**
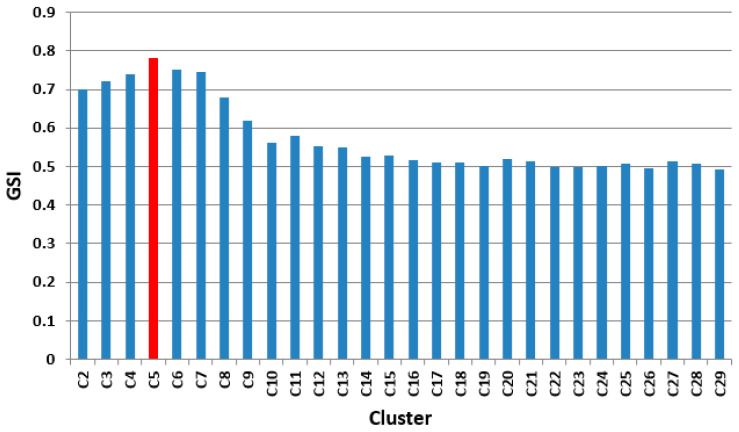
GSI values for cluster ranges between C2 and C29.

**Figure 11:**
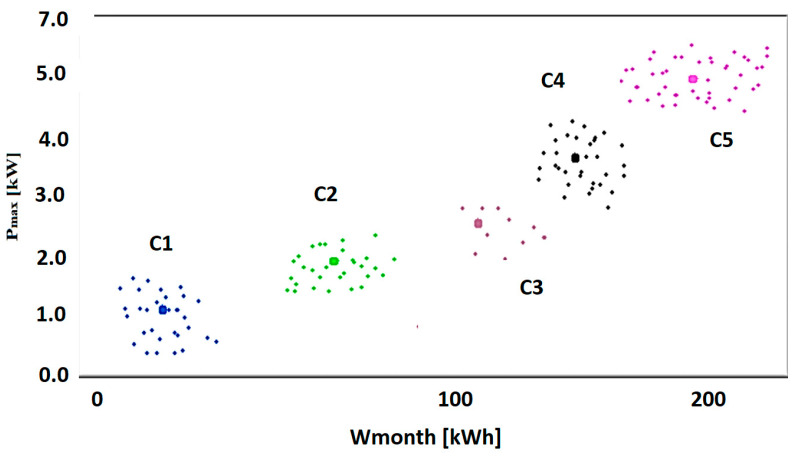
Graphical representation of clusters for the case $K_{optim}=5$.

Then, for each *K* = 2, …, 29, the *K*-averages technique was applied. For the clustering processes initiated for the *K* value of the number of clusters, the quality of the grouping process was estimated by calculating the global silhouette index (GSI). The values of the shape index obtained for each *K* = 2, …, 29 are represented in Figure 10. This grouping is the best result of the clustering technique since the GSI has the greatest value for *K* = 5. The results for the global silhouette index (GSI = 0.775) show that the clustering technique was quite effective. The graphical representation of the clusters obtained in the case $K_{optim}=5$ is illustrated in Figure 11.

The characteristic variables represented by the mean values (m) and the dispersion (σ) corresponding to the maximum load (P_max_) and the monthly energy (W_month_) for each cluster (consumption category) are summarized in Table 1.

**Figure 12:**
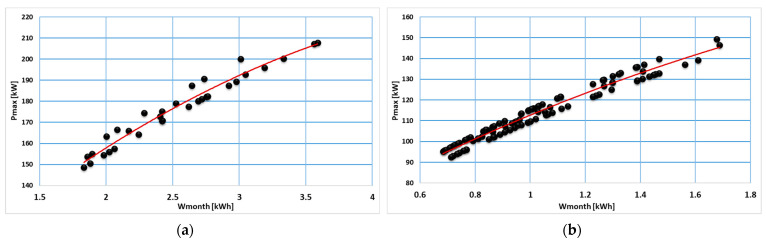
Regression models correlated to consumption class C1 (**a**) and C2 (**b**).

**Figure 13:**
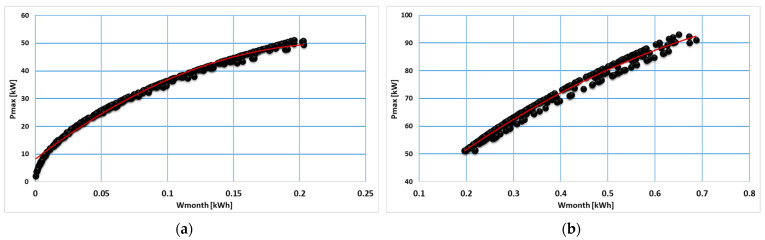
Regression models associated with consumption class C3 (**a**) and C4 (**b**).

**Figure 14:**
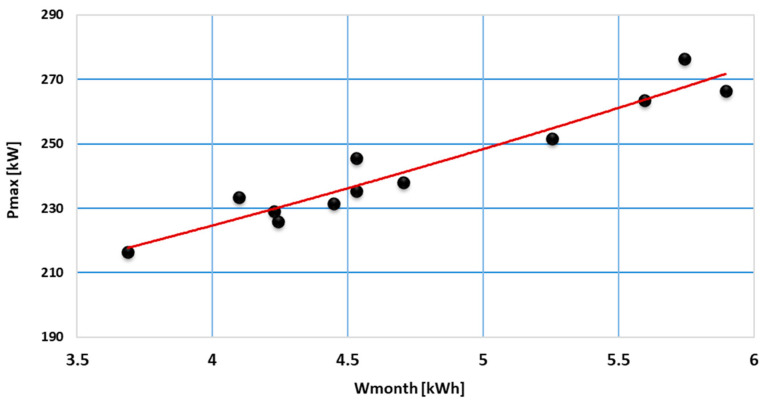
Regression models associated with consumption class C5.

The analysis of data from each consumption category indicated a correlation between the maximum load and the monthly energy consumption that can be mathematically modeled by applying regression models. In Figure 12, Figure 13 and Figure 14, regression models are represented based on first- and second-degree polynomials for every consumption class.

Regression models based on the degree II polynomial led to a better approximation than the degree I polynomial. Thus, regression models based on the degree II polynomial were adopted to determine the P_max_ (W_month_) dependence; coefficients of the regression models determined for each consumption class are summarized in Table 2.

In Stage 4, for all consumers in the test base, the maximum load is estimated by applying the regression model of the consumption class associated with every consumer, depending on the monthly energy consumed.

**Figure 15:**
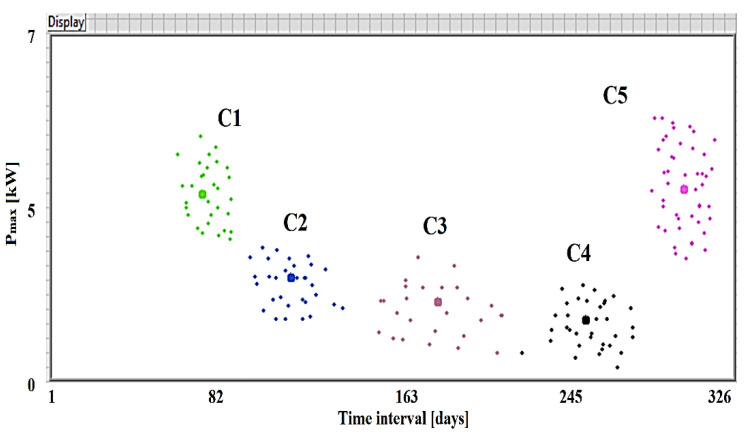
Real and estimated maximum load values of the clustering testing base (C1–C5 represent the clusters used for the analysis).

Figure 15 presents the real and forecasted values associated with the maximum consumer tasks within the test base, assembled according to the consumption class.

**Figure 16:**
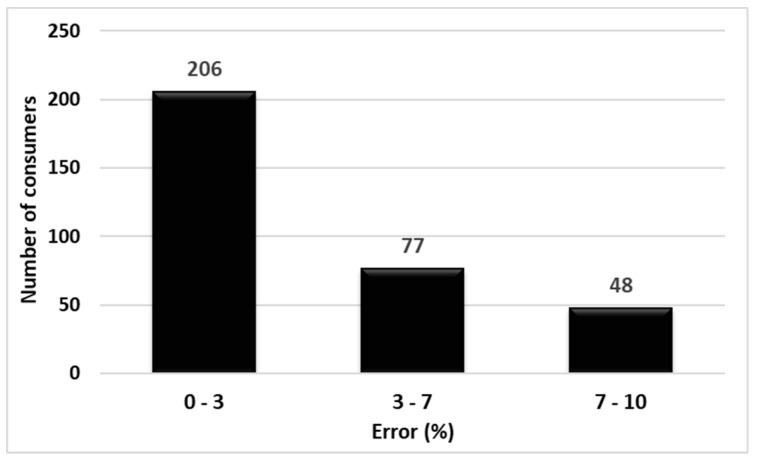
Classification of maximum load forecast errors for testing base consumers.

Of the 331 testing base consumers, 206 (approximately 62%) forecast errors were ≤3%, 77 (approximately 23%) errors were between 3 and 7%, and 48 (approximately 15%) errors were between 7 and 10% (Figure 16); thus, the average estimation error in the testing base was 4.01%. This value is reasonable in the context that most consumers do not have permanent monitoring.

### 3.2. Simulation of a Load of MV/LV Distribution Transformers by Clustering Procedure Application

The future power supply networks of electric vehicles will be powered by medium and low-voltage networks.

The following algorithm is suggested for simulating the load of the distribution transformers from the MV/LV transformation stations [40].

Phase 1. Database: representative consumers with smart meters will be sorted out from the database. Typical load diagrams will be recorded for every consumer, then, the main characteristic variables will be detached, i.e., minimum (P_min_) and maximum active power (P_max_), daily energy (W_z_), and consumption class.

Phase 2. Pre-processing load diagrams: all records involving missing data or values will be excluded or subjected to processing. After being pre-processed and reduced, the results will be applied to obtain the classification into consumption categories (clusters) using the clustering procedure.

Phase 3. The division into consumption macro-categories: the database with the records of load schedules will be split into clusters described by the consumer’s type: residential, commercial, and industrial.

Phase 4. Clustering procedure: a clustering technique will be applied for load-type profile determination to determine the optimum results. In the end, a typical load profile for each consumption class will be obtained by applying the average of the hourly values for the load diagrams.

Phase 5. Determining the load profiles: a typical load profile will be attributed to every consumer’s class, depending on their consumption class.

Phase 6. Estimating the load of the MV/LV transformer: a simulation protocol will then be proposed based on the Equation [40]:

(16) $$P^{h}=\sum_{k=1}^{C_{k}}n_{k}W_{med\;k}p_{k}^{h}+\sqrt{\sum_{k=1}^{C_{k}}n_{k}{\left(W_{med\;k}\sigma_{k}^{h}\right)}^{2}\;\;},h=1,\ldots .,24\left[kW\right]$$

where:

$P^{h}$ is the MV/LV transformer load from the transformer station at *h*, (kW);
$n_{k}$ is the consumer number, where *k* is the consumption class;
$W_{med\;k}$ is the average energy consumption, where *k* is the consumption class, (kWh);
$p_{k}^{h}$ is the hourly coefficient of transformation for energy consumed by the consumers, (kW/kWh);
$\sigma_{k}$ is the standard deviation of the power distribution necessary to the cluster consumption (kW/kWh);
$C_{k}$ is the cluster number (consumption class) correlated to the consumer’s feed.

The weight center technique was applied to evaluate the load-type profiles through a database containing 180 load curves registered by smart meters from residential consumers in a distribution system from the LV pilot.

To evaluate the representative load profiles, the gravity center was applied, taking into consideration the scattering of the LV pilot located in a region from Romania [40]. Every load graph is set by 48 hourly values correlated with consumer behavior over one day. Missing load curves or abnormal values of zero throughout the day must be eliminated from the procedure, with only 144 consumers remaining eligible. The clustering procedure resulted in five consumption classes (clusters) (Table 3).

**Figure 17:**
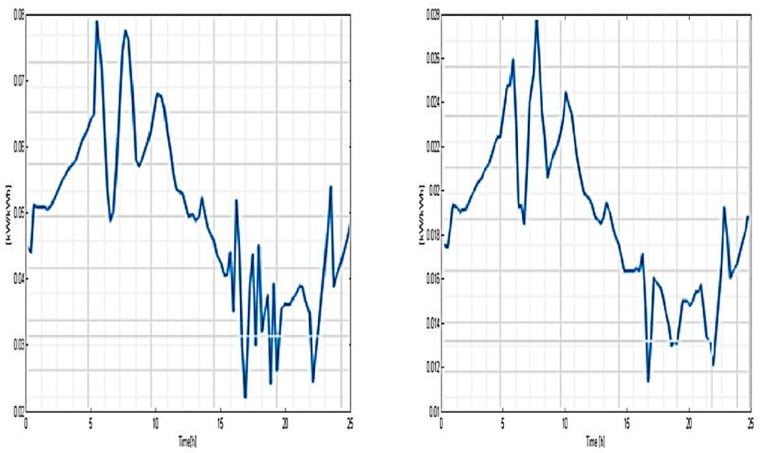
Load-type profile related to C1 and C2 consumption classes.

**Figure 18:**
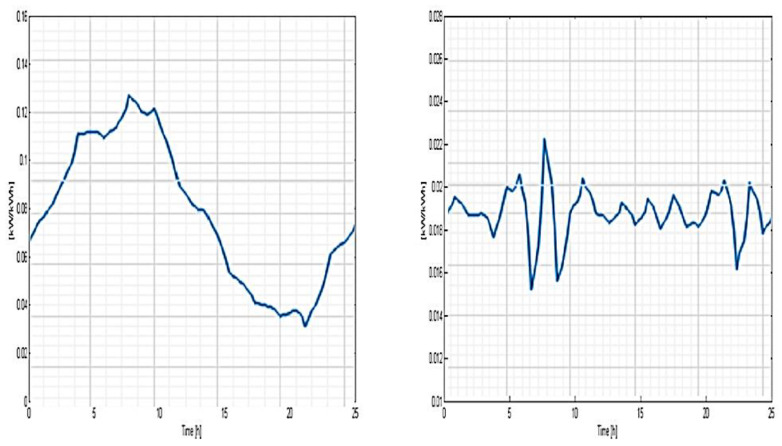
Load-type profile related to C3 and C4 consumption classes.

**Figure 19:**
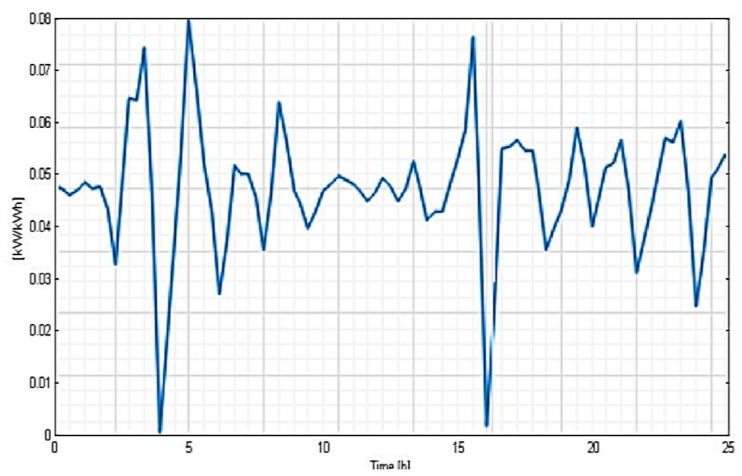
Load-type profile related to C5 consumption class.

**Figure 20:**
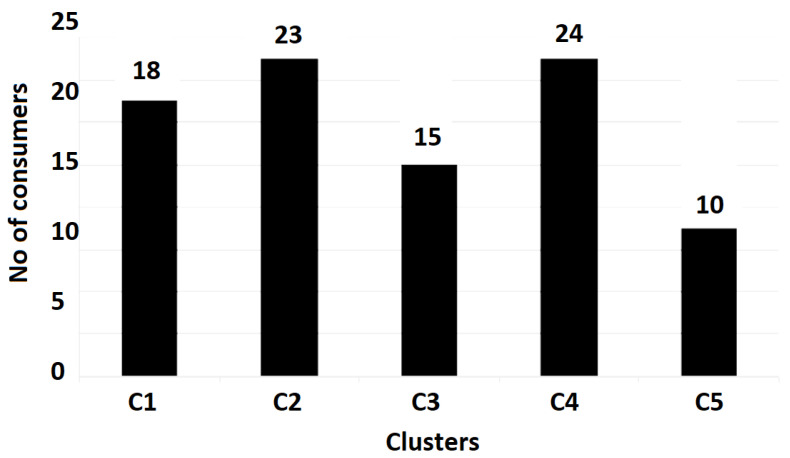
Consumer associations resulting from the testing base.

It can be observed that the most representative class of consumption is C5 (50% of the total consumer number), the least representative being C2 (only 3.5%). The load-type profiles correlated to every cluster (consumption class) are depicted in Figure 17, Figure 18 and Figure 19 and the consumer distribution is presented in Figure 20.

## 4. Discussion

Numerical simulations related to the methodologies, algorithms, and calculation programs developed in this paper have shown that the intelligent distribution of consumers in Smart Grid distribution systems can help smooth the charging curve that can lead to lower electricity prices and facilitate the integration of renewable energy sources, resulting in a much safer and more economical operation of Smart Grid networks. The authors in [37,38] perform an analysis of data extraction techniques from the perspectives of different technical approaches to achieve consumer profiles using direct clustering, indirect clustering, clustering evaluation criteria, and customer segmentation. The article [39] presents an approach to the consumer profile from the perspective of time series, and in [38], the issue discussed is approached with the Bayes model and k-means clustering. As can be seen from the literature, clustering algorithms are frequently used in the energy field for profiling consumers. The method proposed in this article combines grouping algorithms by clustering techniques and evaluation criteria for the clustering results using the regression algorithm with second-order polynomials (logistic regression). By separating consumer behaviors, the relationship between them can be simplified. The use of hierarchical clusters (hierarchical classification) can significantly reduce the influence of external factors (e.g., region, weather, time, day, and social activities) on classifier performance. The results of the case study showed that the model proposed in this paper achieves a better classification of electricity consumption. In addition, the technique presented in this article contributes to an overall improvement in the profiling of consumers, as the proposed method achieves a better classification using fewer training samples. The performance of the results presented suggests that the proposed data-based model can be used as an effective tool in real-time. The idea of load demand variability is key information for the load monitoring control unit, thus the proposed task prediction models will help energy management. Based on a more accurately forecasted load demand, different optimization techniques for demand response applications can be developed. In addition, the classification of the model proposed in this paper depends on the completeness and reliability of the data. By combining these two algorithms in the next stage, we plan to develop a supervised machine learning algorithm that will automatically determine the profile of consumers based on historical data and data acquired in real-time (data mining).

## 5. Conclusions

Following the proposed study that formed the basis of this article, some conclusions can be made:

o Urban areas have significant issues in several areas, including the economy, water supply, energy, buildings transit, environmental protection, and basic services as a result of the phenomenon known as “global urbanization”.
o Municipalities are encouraged to employ smart ideas and try various smart infrastructure approaches in order to address these problems, thereby becoming the future smart cities or “Smart Cities”.
o Urban transportation issues are a key component of the Smart City idea, and the approximately 7.2 million electric passenger and freight vehicles demonstrate that electrification of the transportation sector is the undeniable future of mobility.
o The restrains in regulations on the use of conventional fossil fuels in Europe and China caused the automotive industry to quickly realign to multiple EV and BEV models, with more than 442 new products being available at this moment, leading to an 87% drop in Lithium-Ion battery prices, per kWh, between 2010 and 2019. These factors all have contributed to the rapid growth of the number of EVs. When considering the above-mentioned factors, electric vehicles have emerged as one of the primary solutions for decarbonizing the transportation industry and using renewable energy sources to generate electricity. Their impact on electrical networks, however, cannot be disregarded. The quest for low-emission mobility around the globe is expected to drive a major increase in the electrification of road transportation in the next decades. The volume of the world’s electricity demand may shift as a result of the rise in electric vehicles, posing serious problems for the infrastructure supporting electricity production, transmission, and distribution.
o The integration of too many EVs will significantly impact the electric power systems; however, by coordinating EV charging, flexibility services in the electric power network can be achieved, and the required investments in infrastructure can be kept to a minimum level.

Future study directions suggested to continue the research outlined in this paper include:

- Participation of EVs or charging stations equipped with converters that use power electronics in reactive power regulation services for EVs.
- Offering support services by coordinating EV charging via LV power grids.
- Variations in the voltage level caused by EV fleets since it is equal to the electrical charge throughout the steady state operation or photovoltaic renewable energy sources while supplying energy to the grid.
- Analysis of hybrid solutions utilizing battery energy storage systems for the necessary integration of ultra-fast charging stations with capacities of up to 350 kW in metropolitan electricity networks.

## Figures and Tables

**Table 1:** Statistical variables of the characteristic variables associated with the consumption categories.

Consumption Class	Number of Consumers	P_max_ (kW)		W_month_ (kWh)	
		m	σ	m	σ
C1	31	1.51	0.32	192.20	13.66
C2	104	1.06	0.13	115.62	11.83
C3	387	0.43	0.09	30.70	12.93
C4	279	0.87	0.17	88.64	11.83
C5	12	4.78	0.92	252.96	19.70

**Table 2:** Regression model coefficients associated with consumption categories.

Consumption Class	a (×10^−5^)	b (×10^−3^)	c
C1	13	22	2.6
C2	6.2	3.1	0.7
C3	7.3	0.023	0.00008
C4	8.2	0.45	0.027
C5	15	105	17

**Table 3:** Consumption class characteristics.

Consumption Class	Number of Consumers	P_max_ (kW)		P_min_ (kW)		W (kWh)	
		m	σ	m	σ	m	σ
C1	15	0.21	0.02	0.06	0.01	3.41	0.82
C2	5	0.51	0.05	0.02	0.02	4.35	1.20
C3	22	0.46	0.12	0.03	0.01	2.98	0.54
C4	30	0.04	0.06	0.02	0.03	0.25	0.43
C5	72	0.17	0.06	0.03	0.02	1.95	0.41

## Data Availability

Not applicable.
